# Production of poly-β-1,6-N-acetylglucosamine by MatAB is required for hyphal aggregation and hydrophilic surface adhesion by *Streptomyces*

**DOI:** 10.15698/mic2018.06.635

**Published:** 2018-02-12

**Authors:** Dino van Dissel, Joost Willemse, Boris Zacchetti, Dennis Claessen, Gerald B. Pier, Gilles P. van Wezel

**Affiliations:** 1 Molecular Biotechnology, Institute of Biology, Leiden University, Leiden, The Netherlands.; 2 Division of Infectious Diseases, Department of Medicine, Brigham and Women’s Hospital, Harvard Medical School, Boston, MA, USA.

**Keywords:** Streptomyces, hyphal aggregation, adhesion, PNAG, biofilm

## Abstract

Streptomycetes are multicellular filamentous microorganisms, and major producers of industrial enzymes and bioactive compounds such as antibiotics and anticancer drugs. The mycelial lifestyle plays an important role in the productivity during industrial fermentations. The hyphae of liquid-grown streptomycetes can self-aggregate into pellets, which hampers their industrial exploitation. Here we show that the Mat complex, which is required for pellet formation, catalyzes the synthesis of extracellular poly-β-1,6-*N*-acetylglucosamine (PNAG) in the model organisms *Streptomyces coelicolor *and *Streptomyces lividans*. Extracellular accumulation of PNAG allows *Streptomyces* to attach to hydrophilic surfaces, while attachment to hydrophobic surfaces requires a cellulase-degradable extracellular polymer (EPS) produced by CslA. Over-expression of *matAB *was sufficient to restore pellet formation to *cslA* null mutants of *S. lividans*. The two EPS systems together increase the robustness of mycelial pellets. These new insights allow better control of liquid-culture morphology of streptomycetes, which may be harnessed to improve growth and industrial exploitation of these highly versatile natural product and enzyme producers.

## INTRODUCTION

Members of the genus *Streptomyces* are of great industrial and medical importance for their ability to produce a plethora of natural products, including antibiotics, anticancer agents and immunosuppressants 1,2, and many industrially relevant enzymes, such as polysaccharide hydrolases and proteases 3. Streptomycetes exhibit a complex multicellular life cycle 4. After germination of a single spore, a complex vegetative mycelium is formed following a process of apical growth and branching of the hyphae 5. The centrally located hyphae undergo a developmental cycle leading to the formation of reproductive aerial hyphae, which are erect and segment into long chains of spores 1. This process is accompanied by the lysis of nearby positioned mycelia 6, thereby liberating nutrients required for aerial growth and sporulation 7 and the production of antibiotics 8.

In submerged cultures, many streptomycetes form mycelial aggregates often referred to as pellets. Because of their dense architecture, which among others causes significant mass-transfer limitations, pellets are undesirable from the perspective of industrial process design. However, antibiotics are often produced more efficiently by mycelial clumps 9,10. Pellet architecture depends on genetic and environmental factors, like septum formation 11,12, shear stress 13, pH 14 and cell-wall fusion 15. Another important factor is the extracellular matrix, which embeds the mycelia 16. Omnipresent in all biofilms, the matrix facilitates cell signaling 17 and allows retaining liberated nutrients 18, while it also plays a major role in growth and morphogenesis 19,20,21. The *Streptomyces *extracellular matrix is a complex mixture of biopolymers containing DNA 22, amyloid proteins 23 and multiple polysaccharides 24. One polysaccharide is produced at the hyphal apex by CslA and tailored by the combined efforts of GlxA and DtpA 23,24,25,26. CslA likely produces a cellulose- or chitin-like polymer, as the polymer is specifically stained by calcofluor white, which is specific for β-1,4 glycans 24. CslA plays a role in the anchoring of fimbriae-like structures attached to the hyphae of *Streptomyces coelicolor*, facilitating the attachment to polystyrene surfaces by working in unison with the amphipathic chaplin proteins 23*.* This attachment was negated by the addition of cellulases, which releases the fimbriae from the cell surface. Interestingly, removal of *cslA *and *glxA* from wild-type *S. lividans* or *S. coelicolor* inhibits pellet formation in liquid-grown cultures 24,25. Pellets are formed by the self-aggregation of the mycelia in a submerged environment and share many characteristics with surface-grown colonies, including the lysis of internally positioned mycelia, with the co-location of antibiotic production 27. Transcriptomics analysis has also shown the activity of developmental genes in pellets of *S.* *coelicolor,* even though sporulation is absent in liquid grown cultures 28.

Recently we discovered a novel locus encompassing the *matA* and *matB* genes*, *which likely encode biosynthetic enzymes for an extracellular polymeric substance (EPS) and are also required for pellet formation 29. Deletion of *matAB* results in a submerged morphology strikingly similar to that of *cslA* mutants 29. Both loci are also required for spore aggregation, which contributes to the size heterogeneity of mycelia in liquid-grown cultures 30. In this study we show that the product of MatAB is a major component of the extracellular matrix and identify the exo-polysaccharide as poly-β-1,6-*N*-acetylglucosamine (PNAG). Attachment assays show that PNAG plays a different function in aggregation than the cellulose-like EPS produced by CslA/GlxA. The results are a major step forward in understanding how the extracellular matrix of *Streptomyces* facilitates adhesion and how it controls morphogenesis during submerged cultivation.

## RESULTS

### The Mat enzymes facilitate the formation of a granular layer on the outside of the hyphae

**Figure 1:**
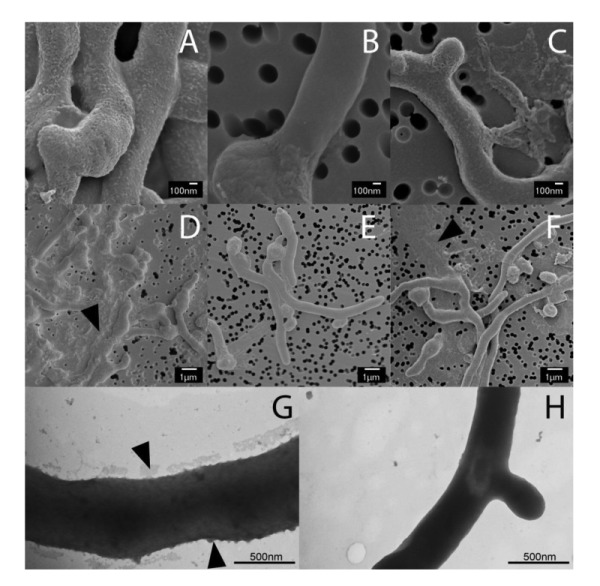
FIGURE 1: Electron micrographs revealing at Mat-dependent extracellular matrix. Scanning electron micrographs of young vegetative mycelium **(A-F)** show an abundance of extracellular material covering the hyphae of wild-type *S. lividans* **(A)** and between hyphae (**D**, indicated by arrow). This extracellular material was also present in the *cslA* null mutant (**C** and **F**, indicated by arrow), but was absent in *matAB* null mutants (**B** and **E**). Negatively stained hyphae with tungsten acid, specific for polymeric substances, revealed a scabrous outside coating in wild-type hyphae **(G)** that is absent in the *mat *mutant **(H)**. All strains were grown for 8 h in TSBS media in shake flasks.

Previous studies have shown that the *mat* genes are required for mycelial aggregation and pellet formation in submerged cultures of *S. coelicolor *and *S. lividans *29. Genome annotation suggested that *matB* encodes an extracellular polysaccharide synthase. To shed more light on the Mat-dependent mechanism, we investigated the cell surface of *S. lividans* at high resolution by cryo-scanning electron microscopy (SEM) (Fig. 1). This revealed a surface layer that decorates the entire outer surface of wild-type hyphae (Fig. 1A), which was absent in the *matAB* null mutant (Fig. 1B). Although the Mat proteins are expressed throughout growth 30, the Mat-dependent polymer was most apparent in young mycelia. Transmission electron microscopy (TEM) of Tungsten acid-negative stained cells, which images electron dense polymeric surface structures, further highlighted the Mat-dependent extracellular layer decorating wild-type hyphae (Fig. 1G). Between the hyphae a deposit of extracellular matrix could also be observed by SEM (Fig. 1D). Conversely, the hyphae of the *matAB* double mutant have instead a smooth surface, observed both with SEM (Fig. 1B) or negative staining in the TEM (Fig. 1H). We also failed to detect any extracellular material between the hyphae of *matAB* mutants (Fig. 1E). The *cslA* null mutant retained the extracellular layer on the cell surface as well as the adhesive material between the hyphae (Fig. 1C and H), again suggesting that this EPS is produced independent of CslA.

### Bioinformatic analysis of the MatA and MatB enzymes

Bioinformatics analysis of MatA failed to identify known protein domains. MatB contains two functional domains, namely an intracellular glycosyltransferase type 2 (GT2) domain and an extracellular type 4 carbohydrate esterase (CE4) domain, connected by a predicted transmembrane helix. Sequences of glycosyltransferases and carbohydrate esterases were extracted from CAZy, which catalogs enzymes with characterized function, and assembled in a local database for Blast analysis. The glycosyltransferase domain of MatB returned PgaC from *E. coli* as the top hit (Table S1). *E. coli* *pgaC* encodes a glycosyltransferase that synthesizes PNAG 31. The next nearest homologs were enzymes with the same function in *Acinetobacter baumannii,* *Staphylococcus epidermis, Aggregatibacter actinomycetemcomitans and Actinobacillus pleuropneumoniae*, all with similar scores.

A similar blast comparison with the MatB carbohydrate esterase domain returned PgdA (BC_3618), a peptidoglycan N-acetylglucosamine deacetylase from *Bacillus cereus* as nearest characterized homologue (Table S1). Other top hits include a chitin deacetylase from *Caldanaerobacter subterraneus *and NodB proteins from *Rhizobium* species, all with similar scores. Interestingly, these enzymes all act on 1,4-linked oligo-chitin like substrates, in contrast to poly-β*-*1,6-N-acetylglucosamine glycosyltransferases. Taken together, bioinformatics analysis predicted that MatB catalyzes the formation of a poly-*N*-acetylglucosamine, with either a (1,4)- or (1,6)-configuration.

**Figure 2:**
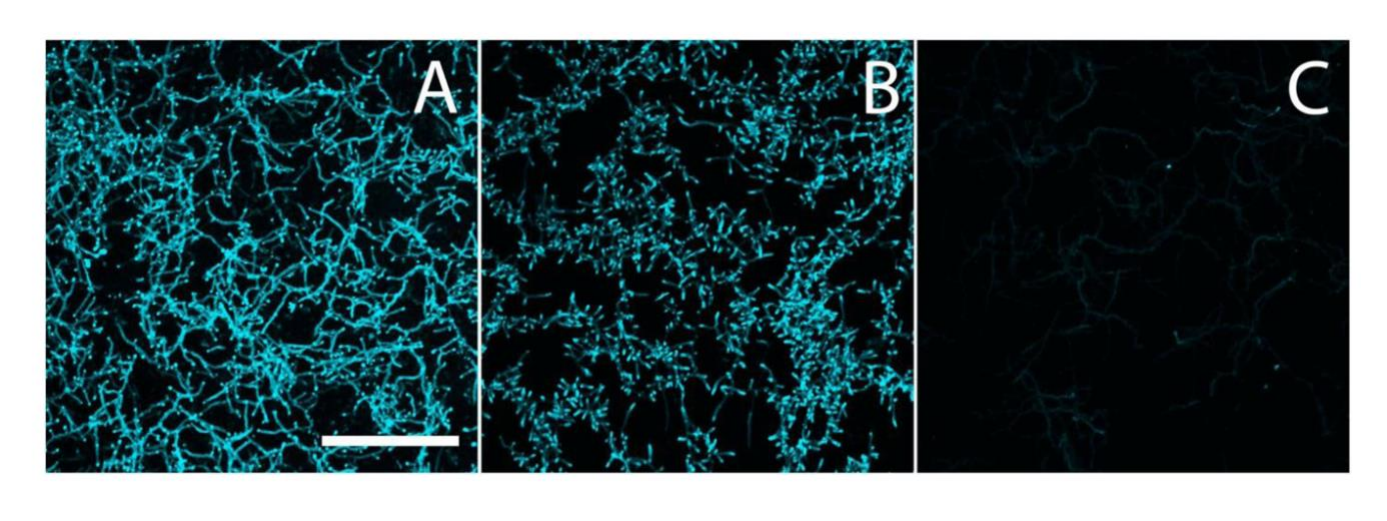
FIGURE 2: Calcofluor white staining to identify β-(1,4)-glycans. *S. lividans ***(A)**, and its *matAB* **(B)** and *cslA* **(C)** mutants were stained with calcofluor white (CFW) to identify the presence of extracellular β-(1,4)-glycans. The staining patterns indicated the presence of β-(1,4)-glycans in both the parental strain and its *matAB* mutant, but not in the *cslA* mutant. Bar, 50 µM.

### Biosynthesis of a PNAG-like EPS by the MatA and MatB enzymes

To analyze if the Mat proteins are involved in the biosynthesis of β-(1,4-) glycans, hyphae of *S. lividans* 66 and its *matAB* null mutant were stained with calcofluor white (CFW), which is specific for glycans in this configuration 32. Apical sites of both wild-type and *matAB* mutant cells were stained with equal efficiency (Fig. 2). This contrasts with the absence of staining in* cslA* null mutants, where the synthesis of β-(1,4)-glycans is impaired 24,25. These data suggest that only the CslA-GlxA system, and not MatA/MatB, produces β-(1,4)-glycans in *S. lividans*.

**Figure 3:**
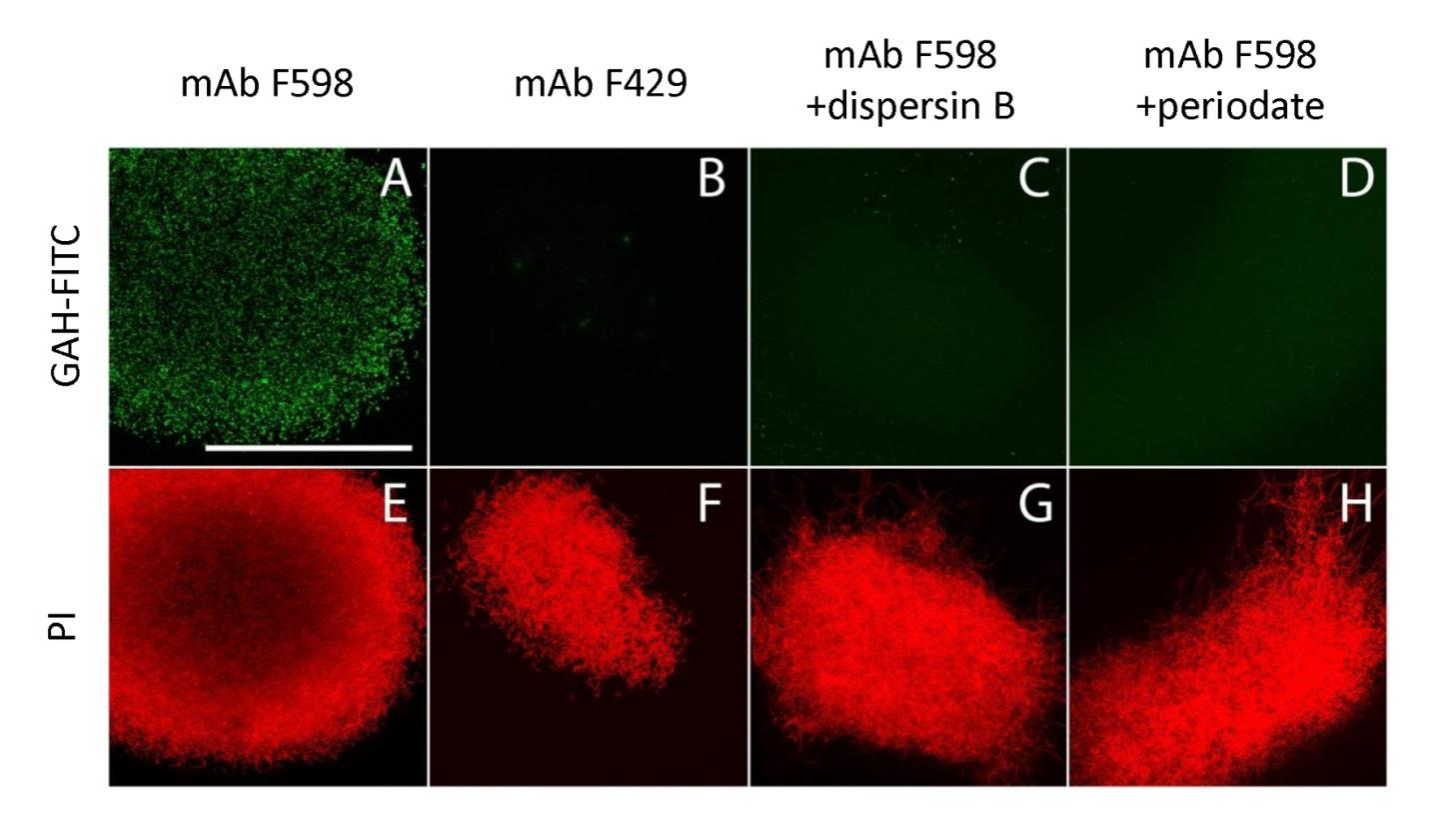
FIGURE 3: Immunofluorescence micrographs of *S. lividans* identifying extracellular PNAG. *M*ycelia from 18 h old cultures of *S. lividans *66 were analyzed for the presence of PNAG with the monoclonal antibody mAb F598 and secondary anti-human IgG Alexa 488 conjugate (green) **(A,C,D)**. As controls for the specificity of the primary antibody we used mAb F429, a monoclonal antibody that binds alginate **(B)**, and samples treated with 50 µg/ml dispersin B **(C)** that degrades PNAG or with 0.4 M periodate **(D)**, which degrades β-(1,6)-glycans. To visualize the hyphae, the DNA was stained with propidium iodide (red) **(E-H)**. Bar, 100 µm.

**Figure 4:**
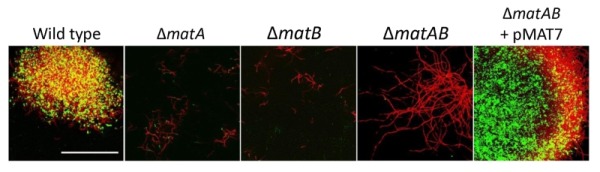
FIGURE 4: PNAG biosynthesis depends on MatA and MatB. Mycelia from 18 h liquid-grown cultures were stained with mAb F598 (green), showing PNAG-specific staining for the wild-type strain *S. lividans* 66, while it was absent in mutants lacking *matA*, *matB *or the *matAB* double mutant. Genetic complementation via introduction of pMAT7 expressing *matA *and *matB* restored PNAG formation to the *matAB* double mutant. To visualize the hyphae, the DNA was stained with PI (red). Colocalization of PI and mAb F598 is shown in yellow. Bar: 50 µm.

To further characterize the product of MatA and MatB, we used monoclonal antibodies (mAb F598) that specifically recognize both intact and deacetylated PNAG 33. Mycelia obtained from 18 h liquid-grown cultures of *S. lividans* 66 were fixed in 4% PVA and incubated overnight with mAb F598. After washing and incubation with a fluorescently labeled secondary antibody conjugate, mounting fluid containing propidium iodide (PI) was added to stain the DNA and samples were then imaged with a confocal fluorescence microscope (Fig. 3). Wild-type cells were strongly stained with mAb F598, indicating the production of a PNAG-like polymer. Co-localization with the DNA stain PI suggests that most PNAG-like molecules are located within the pellet’s structure. Conversely staining with the control antibody mAb F429, which binds alginate 34, did not result in a fluorescent signal (Fig. 3B). Binding of mAb F598 could be negated by the PNAG-specific enzyme dispersin B 35 (Fig. 3C) or by the addition of periodate (Fig. 3D), which degrades 1,6-polysaccharides. Conversely, immunofluorescence microscopy of *matA* or *matB *null mutants or the *matAB *double mutant with the mAb F598 antibody only resulted in background fluorescence. The anti-PNAG signal was restored by the introduction of plasmid pMAT7, which expresses *matAB* from the strong *gapA* (SCO1947) promoter (Fig. 4). To link the Mat-dependent extracellular layer seen by SEM to the presence of PNAG, the mycelia were treated for 2 h with a suspension containing either chitinases, cellulases or dispersin B. Importantly, only dispersin B significantly affected EPS accumulation as visualized by SEM microscopy (Fig. S1). Taken together, these data strongly suggest that the MatAB-dependent extracellular matrix is indeed PNAG and that PNAG is not produced in the absence of MatA or MatB.

### MatAB expression is sufficient for pellet formation

**Figure 5:**
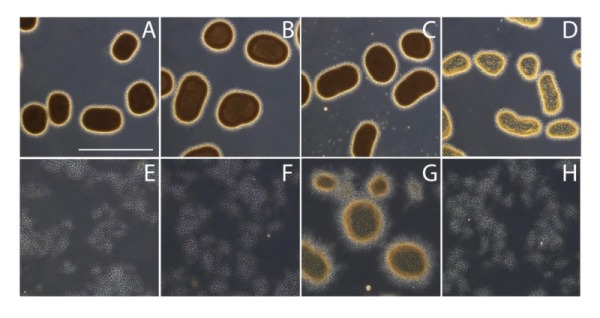
FIGURE 5: Effect of cellulases and dispersin B on mycelial morphology. Light micrographs show *S. lividans *66 **(A)**, *S. lividans *66 treated with 2 U/ml cellulase **(B)**, 100 µg/ml dispersin B **(C)** or both cellulase and dispersin B **(D)**, the *matB* **(E)** and *cslA* mutants **(F)** and the *cslA* mutant harboring pMAT7 without **(G)** or with **(H)** added dispersin B. All strains were grown in TSBS medium for 24 h at 30^o^C. The effects on morphology were visualized by wide-field microscopy. Bar, 500 µm.

As mentioned before, genomic disruption of either *cslA* or *matB* prevents pellet formation in shaken liquid-grown cultures, transforming the morphology in a strikingly similar fashion (Fig. 5A, E-F). Although the Mat-produced PNAG can be enzymatically dispersed by dispersin B (Fig. 3), addition of dispersin B to submerged grown mycelia failed to prevent pellet formation by *S. lividans* (Fig. 5B). Similarly, addition of cellulase, which antagonizes the *cslA* dependent glycan, failed to disrupt pellet formation (Fig. 5C). Simultaneous treatment with cellulases and dispersin B resulted in a slight reduction of the aggregation, reducing the average pellet size, but did not result in the dispersed morphology seen when either *matB *or *cslA *is disrupted (Fig. 5D). Interestingly, introduction of plasmid pMAT7 in the *cslA* mutant resulted in a pelleting phenotype, indicating that *matAB* expression by itself is sufficient for pellet formation (Fig. 5G). This MatAB-driven complementation of pellet formation in the *cslA* mutant could be readily antagonized by the addition of dispersin B, providing further evidence that production of PNAG is indeed the cause of the restored pellet formation (Fig. 5H). These data also indicate that native pellet formation, dependent on both *cslA* and *matAB*, results in a robust structure that cannot be easily antagonized by the addition of degrading enzymes, likely by making (parts) of the matrix inaccessible to the enzymes.

### Mechanistic insight into *matA* and *matB *in relation to *cslA*

**Figure 6:**
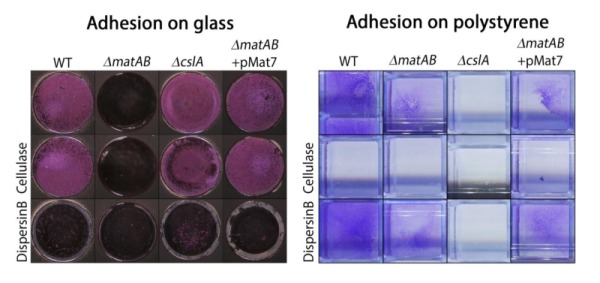
FIGURE 6: Adhesion assays visualizing EPS-mediated attachment to glass and polystyrene. Attachment of *S. lividans* 66 to glass was antagonized by treatment with dispersin B (50 µg/ml) or by deletion of *matAB*, while addition of cellulase (0.2 U/ml) or deletion of *cslA* had no effect. The opposite was seen for attachment to polystyrene, which was antagonized by cellulase or by deletion of *cslA* and not by dispersin B or deletion of *matAB*. The ability to attach to glass was restored to *matAB* mutants by the introduction of plasmid pMAT7.

To further assess the contribution of the two different EPSs to hyphal aggregation and pellet formation, we investigated the affinity of the hyphae for hydrophobic and hydrophilic surfaces, via adherence assays on glass and polystyrene, respectively. Attachment to glass depended on *matA* and *matB*, and was not visibly reduced in *cslA* mutants as compared to that of the parental strain (Fig. 6, left). Conversely, attachment to polystyrene was mostly dependent on *cslA*, and was less affected in *matA *and *matB* mutants (Fig. 6, right). Quantification of attachment by measuring cell-attached crystal violet spectrophotometrically, further confirmed the distinct roles for these genes (Fig. S2)*.* This indicates that the EPS produced by CslA plays a dominant role in adherence to hydrophobic surfaces, while the PNAG produced by MatAB is particularly relevant for adherence to hydrophilic surfaces. This not only shows that the two EPSs have distinct non-redundant roles in aggregation, but also suggests that pellet formation in shaken liquid cultures depends on both hydrophilic and hydrophobic adhesive forces.

## DISCUSSION

Members of the multicellular filamentous genus *Streptomyces* have an innate ability to self-aggregate in liquid-grown cultures. Because the multicellular mycelia are already connected, relatively little is needed for the neighboring hyphae to aggregate and start the formation of dense pellets. This propensity to self-aggregate into pellets contrasts with cells that undergo planktonic growth 4,36. Streptomycetes are the major producers of antibiotics and also produce a plethora of other medically relevant natural products as well as many industrial enzymes, and there is a strong correlation between the mycelial morphology in liquid-grown cultures and productivity 37. Enzyme production is typically favored by mycelial fragmentation 9,29, and it was shown that the Tat protein translocation machinery is located near apical sites 38. Conversely, production of erythromycin and actinorhodin requires a minimum mycelial pellet size 9,10. Therefore, it is important to understand the factors that control liquid-culture morphology.

In this study we investigated two major extracellular polysaccharides that play a crucial role in the ability of *Streptomyces lividans* to adhere. A polysaccharide produced by the recently discovered MatAB proteins was found to be poly-β-1,6-*N*-acetylglucosamine (PNAG). Extracellular polysaccharides in the matrix of bacteria with a planktonic lifestyle play a major role in biofilm formation. Although many types of exo-polysaccharides are employed by different bacteria 39,40, pathogenic bacteria often produce PNAG to adhere to their host 41,42,43,44. The soil bacterium *Bacillus subtilis* also produces PNAG, suggesting that this EPS is abundantly present in natural matrices 45.

The *mat* gene cluster resembles the PNAG biosynthetic gene cluster *icaADBC* from *S. epidermidis*, with the bifunctional MatB likely corresponding functionally to both IcaA and IcaB, forming an intracellular glycosyltransferase domain and an extracellular oligo-deacetylase domain, respectively. SCO2961, which is located directly downstream of *matB*, is an orthologue of *icaC* that might also play a role in formation of the mature polymer. The function of MatA is unclear as it lacks known functional domains, but as deletion of the *matA* gene reduces hyphal aggregation it may assist in efficient polymerization of the EPS, similarly to IcaD 46.

The observation that deletion of either* cslA* or *matAB* results in the disintegration of pellets, suggests a certain degree of interdependence between the two systems, and also that each system is required for pellet formation. Although this cannot be ruled out completely, our data show that pellet formation can be restored to *cslA* mutants by the overexpression of *matAB*, strongly suggesting that sufficiently high production of PNAG can compensate for the absence of the cellulase-like EPS produced by CslA. This is supported by the observation that while pellet formation by wild-type mycelia are unaffected by the addition of dispersin B or cellulases, dispersin B is sufficient to disperse pellets of *cslA* mutants overexpressing *matAB*. This suggests that although PNAG alone is sufficient, both EPSs contribute to the robustness of the pellet structure. The simultaneous addition of both dispersin B and cellulases, which should degrade both EPSs, was not sufficient to fully degrade the EPSs, as the mycelia still showed the ability to aggregate. A possible explanation may be the different roles that both systems play in maintaining the integrity of the pellet. The adhesion assays described above showed different roles of the two EPS systems. PNAG biosynthesized by MatAB mediates adhesion to a (hydrophilic) glass surface, a fast process that occurs in overnight cultures. This is supported by our earlier work showing that *matAB* is perhaps more important for the aggregation of germlings than the cellulose-like matrix produced by CslA 30. Adhesion mediated by the CslA-dependent EPS to the hydrophobic polystyrene is slow, requiring a week-long assay. This is likely the result of a more complex system, which likely also involves the developmentally regulated amphipathic chaplin proteins 23. Chaplins also play a role in pellet formation, which is yet poorly understood. The chaplin-based hydrophobic forces might contribute to strengthening of the pellet. Earlier work indicated that the EPS produced by CslA/GlxA plays a role in stabilization of the tip complex 24, which might explain its pleiotropic involvement in multiple systems throughout the life cycle. We hypothesize that aggregation by PNAG may also depend on proper organization of the tip complex and the presence of the cellulose-like EPS. High resolution spatial co-localization studies of CslA/GlxA, the chaplins and MatAB in native pellets, should shed more light on the involvement and interplay of these systems in the control of the mycelial architecture. This is currently being investigated in our laboratory.

Understanding how hyphal aggregation and pellet formation are controlled also brings us an important step closer to controlling the morphology of streptomycetes in liquid-grown cultures, which is highly relevant for tuning the morphology to product formation during industrial fermentations 37,47 Antibiotics such as erythromycin (produced by *Saccharopolyspora erythraea*) and actinorhodin (by *S. coelicolor*) are produced solely when a minimum pellet size is achieved, while enzyme production is typically favored by fast growing and fragmenting hyphae 9,10. Previous genetic approaches were followed to tune mycelial morphology. Over-expression of *ssgA,* which controls hyphal morphogenesis and activates cell division 11,12, effects fragmentation of the hyphae by enhancing cell division, resulting in increased growth and enzyme production rates 9. However, a drawback to this approach is the major effect of SsgA on the cell cycle, with enhanced sensitivity to shear stress as a result. In this respect morphological engineering targeting extracellular glue-like substances such as PNAG and the cellulose-like EPS, offers a very attractive alternative, as the effects on the internal physiology are likely minimal. Thus, besides their high relevance for our ecological understanding of how streptomycetes grow and attach to surfaces in their natural environment, the insights gained by this work may also help to develop novel technologies that improve growth and productivity of streptomycetes.

## MATERIALS AND METHODS

### Bacterial strains and plasmids

The bacterial strains and plasmids used in this study are listed in Table S2. *E. coli* JM109 48 was used as a routine host for plasmid construction. The native *matAB* locus (SCO2963 and SCO2962) and *gapA *(SCO1947) promoter region were PCR-amplified from the *S. coelicolor* genome as described using primers SCO2963_F, SCO2962_R and PSCO1947_F, PSCO1947_R respectively (Table S1). The *matAB* locus was cloned as an EcoRI/BamHI fragment into the integrative vector pSET152 49 and the promoter region was placed in front of the *matAB *locus as an EcoRI/NdeI fragment, resulting in construct pMAT7. Conjugative plasmid transfer to *Streptomyces* was done using *E. coli* ET12567 50 harboring pUZ8002 as the host 51.

### Culturing conditions

Streptomycetes were grown in shake flasks with a coiled stainless-steel spring in 30 ml tryptic soy broth (Difco) with 10% sucrose (TSBS). Cultures were inoculated with 10^6^ cfu/ml and grown at 30^°^C. To assess growth in the presence of hydrolytic enzymes, strains were grown in 96-well plates where the agitation was facilitated by a Microplate Genie Digital mixer (Scientific Industries, USA) set to 1400 rpm, which was found to reproduce native morphologies at a micro scale 52. Dispersin B (50 µg/ml), cellulase (Sigma Aldrich, C1184) (2 U/ml) or a combination of both were added during growth to degrade EPS. The strains were observed after 24 h of growth by wide field microscopy or processed further for SEM analyses.

### Bioinformatics

The genomes of *Streptomyces coelicolor* A3(2) M145 53 and *S. lividans *66 54 have been published. Protein domains were annotated using the conserved domain search v3.14 55, using default settings. Homology searches were performed using the local Blast+ software v2.2.30 56. A BlastP database was built from the amino acid sequences of all characterized type 2 glycosyltransferases and type 4 carbohydrate esterases listed in the CAZy database (www.CAZy.org). The amino acid sequences were retrieved from the Uniprot database (www.uniprot.org).

### Production and isolation of dispersin B

Dispersin B from *Aggregatibacter actinomycetemcomitans* ATCC 29522 was produced and purified as described 35. The specific activity, determined as the amount of enzyme needed to hydrolyze 1 µmol 4-nitrophenyl-β-D-N-acetylglucosaminide per minute in 50 mM sodium phosphate buffer (pH 5.5) 100 mM NaCl was 570 U /mg protein.

### Calcofluor white staining

The presence of β-(1-4)-glycans was assessed by calcofluor white staining 32. Strains were grown over night in 8-well microscope chambers (LabTech II) in 300 µl TSBS medium. 30 µl calcofluor white (CFW) solution (Sigma Aldrich) was added and after 5 min incubation the samples were imaged on a Zeiss LSM5 Exciter/ Axio observer with a 405 nm laser, a 405/488 nm beam splitter and 420-480 nm bandpass filter 57.

### Immunofluorescence

Immunofluorescence microscopy was performed as described 58, with small adaptations. In short, *S. lividans *66 was grown in TSBS media for 18 h at 30°C. A 50 µl culture aliquot was spotted inside an eight-well microchamber with optical bottom (Lab-Tek, Thermo Scientific). After 15 min the media was removed gently and the cell layer was air dried for 10 min and fixed with 4% paraformaldehyde in PBS for 15 min. After washing samples twice with PBS, monoclonal antibodies against PNAG (mAb F598) or the control which binds alginate (mAb F429) were added to a final concentration of 10 µg/ml in PBS with 0.1% BSA-c (Aurion, the Netherlands) and samples incubated for 16 h at 4°C. The samples were then washed three times with PBS with 0.1% BSA-c and fluorescently-labeled goat-anti-human IgGs (Life Technologies) added to a final concentration of 4 µg/ml and incubated in the dark for 2 h. After washing twice with PBS with 0.1% BSA-c, some PBS with propidium iodide (PI) at a concentration 1 µg/ml was added and the samples were imaged on a Zeiss LSM5 Exciter/ Axio observer with a 488 nm and 543 nm laser, where the emission from the fluorescent antibodies was imaged between 505-545 nm and a 560 nm long-pass filter was used to monitor emission from PI. To remove PNAG, cells were submerged in Tris-buffered saline (pH 6.4) and treaded with dispersin B at a concentration of 50 µg/ml and incubated for 4 h at 37°C or with 0.4 M periodate for 2 h at 37°C. The primary antibody was subsequently added after washing.

### Cryo scanning electron microscopy

Mycelia from cultures grown for 6 h and treated with either PBS (control), PBS containing 50 µg/ml dispersin B, 0.5 U/ml chitinases (Sigma) or 2U/ml cellulases (Sigma). After incubation for 4 h at 37°C the samples were fixed by 1.5% glutaraldehyde and immobilized on isopore membrane 0.8 µm filter discs (Millipore) by pushing the liquid through using a syringe and placing the filter in a filter holder. The discs were cut to size and placed on the SEM target immobilized with Tissue Tek® and quickly frozen in liquid nitrogen slush and transferred directly to the cryo-transfer attachment of the scanning electron microscope. After 10 minutes sublimation at -90°C specimens were sputter-coated with a layer of 2 nm Platinum and examined at -120°C in the JEOL JSM6700F scanning electron microscope at 3 kV as described 45.

### Negative stain TEM microscopy

For negative staining, 5 µl of young mycelium was placed on a copper TEM grid and air dried for 15 min. The cellular material was stained with 3% PTA for 5 min, followed by 5 times washing with milliQ. The samples were placed in a JEOL 1010 transmission electron microscope and observed at 60 kV as described 59.

### Adhesion Assays

Attachment of strains to polystyrene surfaces was tested as described 60. In short 10^6 ^CFUs/ml were inoculated into 4 ml NMMP 51 without polyethylene glycol and casamino acids, using 2% (w/v) mannitol as the sole carbon source. After 5 days at 30^0^C the standing cultures were stained with crystal violet. After washing, the attached cells were quantified by extracting the crystal violet with 10% SDS and measuring the absorption at 570 nm 61. Attachment to glass surfaces was tested in a similar fashion, using glass bottom 96 wells plates (Greiner Bio-One, Austria) and 200 µl NMMP medium without polyethylene glycol, but with 0.5% casamino acids and 2% glucose as the carbon source. These were cultivated overnight at 30^0^C and the attached biomass was quantified as for polystyrene. In case of enzymatic treatment, either cellulase (Sigma) at a concentration of 0.2 U/ml or Dispersin B at a concentration of 50 µg/ml was added to the medium, just prior to inoculation. All measurements are the average of five replicates.

## SUPPLEMENTAL MATERIAL

Click here for supplemental data file.

All supplemental data for this article are also available online at http://microbialcell.com/researcharticles/production-of-poly-%CE%B2-16-n-acetylglucosamine-by-matab-is-required-for-hyphal-aggregation-and-hydrophilic-surface-adhesion-by-streptomyces/.
