# Supplementary material for: Production of poly-β-1,6-N-acetylglucosamine by MatAB is required for hyphal aggregation and hydrophilic surface adhesion by Streptomyces
Source: Microb Cell. 2018 Feb 12;5(6):269–79. doi: 10.15698/mic2018.06.635 (PMC5972031; doi:10.15698/mic2018.06.635)
Supplement: Supplementary file 1 [file mic-05-269-s01.pdf]

SUPPLEMENTARY INFORMATION

belonging to the manuscript

Production of poly-β-1,6-*N*-acetylglucosamine by MatAB is required for hyphal aggregation and hydrophilic surface adhesion by *Streptomyces*

Dino van Dissel[1], Joost Willemse[1], Boris Zacchetti[1], Dennis Claessen[1], Gerald B. Pier[2], Gilles P. van Wezel[1] *

[1] Molecular Biotechnology, Institute of Biology, Leiden University, Leiden, The Netherlands.

[2] Division of Infectious Diseases, Department of Medicine, Brigham and Women's Hospital, Harvard Medical School, Boston, MA, USA

* Corresponding author. Email: g.wezel@biology.leidenuniv.nl; Tel: +31 71 5274310

PO Box 9505, 2300RA Leiden, The Netherlands

Table S1: Similarities between *matB* and characterized glycosyltransferases type 2 and carbohydrate esterase type 4 as found on the Cazy database by BlastP. Based on the annotation of the GT2 and CE4 domain found in *matA* its translated protein sequence was compared with similar typed proteins for which there is experimental evidence as tracked by the CAZY database. Two blast databases were made of the 266 GT2 proteins and the 58 CE4 proteins. The top 10 hits and the scores for both databases are given in the table.

Blastp verus characterized GlycosylTransferase Family 2

Uniprot	*Gene*	*Organism*	*Disciption*	*Score*	*E-value*
P75905	*pgaC*	*Escherichia coli (K12)*	Poly-beta-1,6-N-acetyl-D-glucosamine synthase	157	3E-43
C8YYH7	*pgaC*	*Acinetobacter baumannii*	Poly-beta-1,6-N-acetyl-D-glucosamine synthase	151	2E-41
Q5HKQ0	*icaA*	*Staphylococcus epidermidis*	Poly-beta-1,6-N-acetyl-D-glucosamine synthase	150	6E-41
Q5VJB2	*aagC*	*Aggregatibacter actinomycetemcomitans*	Poly-beta-1,6-N-acetyl-D-glucosamine synthase	140	2E-37
Q5QFG3	*aagC*	*Actinobacillus pleuropneumoniae*	Poly-beta-1,6-N-acetyl-D-glucosamine synthase	137	2E-36
Q84GC8	*hasA*	*Streptococcus equi subsp. zooepidemicus*	Hyaluronan synthase	89	2E-20
O50201	*hasA*	*Streptococcus dysgalactiae subsp. equisimilis*	Hyaluronan synthase	89	2E-20
Q9LJP4	CSLC4	*Arabidopsis thaliana*	Xyloglucan glycosyltransferase 4	87	1E-19
P74165	sll1377	*Synechocystis sp. (PCC 6803)*	Beta-monoglucosyldiacylglycerol synthase	86	3E-19
Q8YMK0	all4933	*Nostoc sp. (PCC 7120)*	Beta-monoglucosyldiacylglycerol synthase	85	4E-19

Blastp verus characterized Carbohydrate Esterase Family 4

Uniprot	*Gene*	*Organism*	*Disciption*	*Score*	*E-value*
Q81AF4	BC_3618	*Bacillus cereus (ATCC 14579)*	Peptidoglycan N-acetylglucosamine deacetylase	140	3E-40
Q8RBF4	*Cda1*	*Caldanaerobacter subterraneus*	chitin deacetylase	133	2E-36
Q81EK9	BC_1960	*Bacillus cereus (ATCC 14579)*	Peptidoglycan N-acetylglucosamine deacetylase	123	2E-33
Q8Y9V5	lmo0415	*Listeria monocytogenes serovar*	Peptidoglycan N-acetylglucosamine deacetylase	125	5E-33
P72333	*nodB*	*Rhizobium sp. (N33)*	Chitooligosaccharide deacetylase	120	7E-33
P02963	*nodB*	*Rhizobium meliloti*	Chitooligosaccharide deacetylase	119	1E-32
Q1M7W8	*nodB*	*Rhizobium leguminosarum*	Chitooligosaccharide deacetylase	118	3E-32
P04339	*nodB*	*Rhizobium leguminosarum bv. viciae*	Chitooligosaccharide deacetylase	118	3E-32
P50355	*nodB*	*Rhizobium sp. (NGR234)*	Chitooligosaccharide deacetylase	118	3E-32
P50354	*nodB*	*Rhizobium galegae*	Chitooligosaccharide deacetylase	118	3E-32

Table S2: Strains used in the study

Strain or plasmid	Description and genotype	Reference
**Strain**		
Streptomyces lividans 66 (1326)	SLP2+ SLP3+	[51]
**Δ*cslA***	*S. lividans* 66 Δ*cslA*	[25, 62]
GAD05	*S. lividans* 66 Δ*matAB*	[29]
GAD06	*S. lividans* 66 Δ*matB*	this work
GAD07	*S. lividans* 66 Δ*matA*	this work
GAD08	*S. lividans* 66 Δ*matAB* + pMAT7	this work
GAD09	Δ*cslA* + pMAT7	this work
**Plasmids**		
**pSET152**	*oriT* RK2, pUC18 replicon, $Apra^R$	[49]
**pMAT7**	pSET152::P_{gapA}-*matAB*	this work

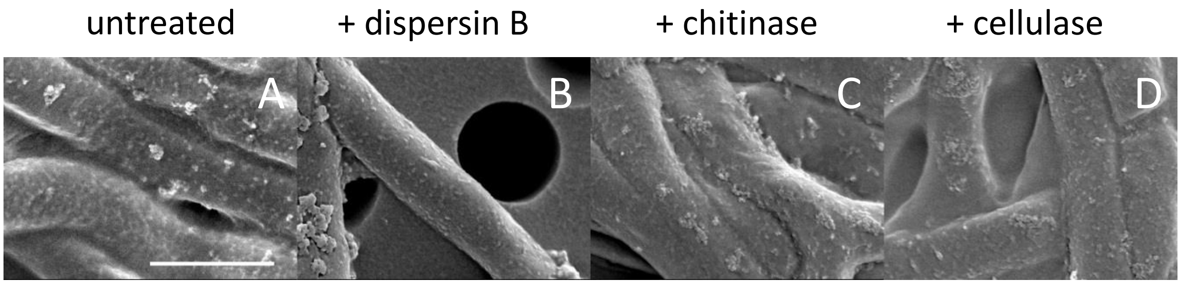

Figure S1. Effect of hydrolytic enzymes on the accumulation of EPS on hyphae of S. lividans 66. Mycelia of wild-type *S. lividans* 66 were treated with either 50 µg/ml dispersin B, 0.5 U/ml chitinases or 2 U/ml cellulases for 4 h. Untreated hyphae were used as the control. The samples were then imaged with SEM to visualize the extracellular matrix. Note that treatment with dispersin B, which degrades PNAG, resulted in a smooth hyphal surface. Bar, 1 µm.

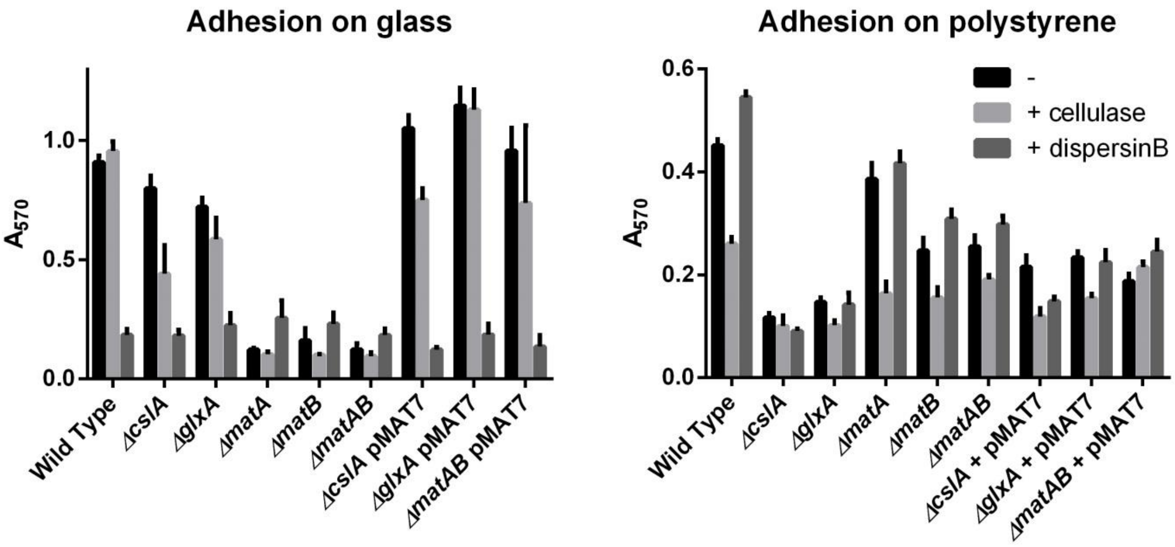

Figure S2. Quantification of attachment to solid surfaces. Surface attachment was quantified for *S. lividans* 66 and its respective *cslA, glxA*, *matA* or *matB* mutants with and without added dispersin B or cellulase. Also shown are the strains harboring the pMAT7 construct, which overexpresses the *matAB* genes. Quantification was performed by staining attached cells with crystal violet and measuring dissolved crystal violet spectrophotometrically at 570 nm. The average and standard deviation of five independent wells are given. Left: surface attachment on glass from overnight growth. Right: surface attachment to polystyrene after 7 days of growth.
